# Enhancement of Cardiac Store Operated Calcium Entry (SOCE) within Novel Intercalated Disk Microdomains in Arrhythmic Disease

**DOI:** 10.1038/s41598-019-46427-x

**Published:** 2019-07-15

**Authors:** Ingrid M. Bonilla, Andriy E. Belevych, Stephen Baine, Andrei Stepanov, Louisa Mezache, Tom Bodnar, Bin Liu, Pompeo Volpe, Silvia Priori, Noah Weisleder, Galina Sakuta, Cynthia A. Carnes, Przemysław B. Radwański, Rengasayee Veeraraghavan, Sandor Gyorke

**Affiliations:** 1 Dorothy M. Davis Heart and Lung Research Institute, College of Medicine, The Ohio State University Wexner Medical Center, Columbus, OH USA; 2 Department of Physiology and Cell Biology, College of Medicine, The Ohio State University, Columbus, OH USA; 3 Division of Pharmacy Practice and Sciences, College of Pharmacy, The Ohio State University, Columbus, OH USA; 4 Division of Pharmacology, College of Pharmacy, The Ohio State University, Columbus, OH USA; 5 Department of Biomedical Engineering, College of Engineering, The Ohio State University, Columbus, OH USA; 6 Department of Biological Sciences, Mississippi State University, Mississippi State, MS USA; 7 Department of Biomedical Sciences, University of Padova, Padova, Italy; 8 Department of Molecular Medicine, University of Pavia, Pavia, Italy; 9 Laboratory of Cell Pathology, Institute RAS, Saint Petersburg, Russia

**Keywords:** Calcium signalling, Calcium and vitamin D

## Abstract

Store-operated Ca^2+^ entry (SOCE), a major Ca^2+^ signaling mechanism in non-myocyte cells, has recently emerged as a component of Ca^2+^ signaling in cardiac myocytes. Though it has been reported to play a role in cardiac arrhythmias and to be upregulated in cardiac disease, little is known about the fundamental properties of cardiac SOCE, its structural underpinnings or effector targets. An even greater question is how SOCE interacts with canonical excitation-contraction coupling (ECC). We undertook a multiscale structural and functional investigation of SOCE in cardiac myocytes from healthy mice (wild type; WT) and from a genetic murine model of arrhythmic disease (catecholaminergic ventricular tachycardia; CPVT). Here we provide the first demonstration of **lo**cal, transient **C**a^2+^ **e**ntry (LoCE) events, which comprise cardiac SOCE. Although infrequent in WT myocytes, LoCEs occurred with greater frequency and amplitude in CPVT myocytes. CPVT myocytes also evidenced characteristic arrhythmogenic spontaneous Ca^2+^ waves under cholinergic stress, which were effectively prevented by SOCE inhibition. In a surprising finding, we report that both LoCEs and their underlying protein machinery are concentrated at the intercalated disk (ID). Therefore, localization of cardiac SOCE in the ID compartment has important implications for SOCE-mediated signaling, arrhythmogenesis and intercellular mechanical and electrical coupling in health and disease.

## Introduction

Store-operated Ca^2+^ entry (SOCE) is the predominant form of Ca^2+^ entry in non-electrically excitable cells, and governs many critical cellular behaviors^1–5^. Although SOCE has been identified in cardiac myocytes^6–11^ it was deemed inconsequential due to the much larger Ca^2+^ fluxes in canonical myocyte excitation-contraction coupling (ECC). However, recent studies linking SOCE to cardiac pathologies such as hypertrophy and arrhythmia^6–9,12,13^ provide impetus for further investigation of this phenomenon.

Canonical ECC in cardiac myocytes is a Ca^2+^ -induced Ca^2+^ release phenomenon, wherein Ca^2+^ from the sarcoplasmic reticulum (SR) is released via ryanodine receptor (RyR2) channels in response to Ca^2+^ entry via L-type Ca^2+^ channels (CaV1.2). The protein machinery of ECC is largely organized into specialized domains within transverse tubules. SOCE represents a complementary process to ECC, and functions to introduce Ca^2+^ into the ER/SR when luminal Ca^2+^ levels within the ER/SR gets depleted^4,14–16^. Thus, SOCE relies on the ER/SR membrane localized Ca^2+^ sensor STIM1 (stromal interaction molecule, isoforms 1 and 2) working in conjunction with store-operated transmembrane Ca^2+^ channels (ORAI; Ca release-activated calcium channel protein, or TRPC; transient receptor potential cation channel, isoforms 1, 4)^17–19^. Current knowledge about cardiac SOCE derives from studies of cell-wide Ca^2+^ changes^6,11^. However, important questions remain about basic functional properties, localization, molecular underpinnings, effector targets and role of cardiac SOCE in health and disease. Importantly, a growing body of evidence links pathological enhancement of SOCE to cardiac disease^6,8^. However, it remains unclear whether and how cardiac SOCE contributes to physiological or pathological intracellular signaling in the presence of much larger Ca^2+^ fluxes involved in canonical ECC.

Here, we provide the first experimental demonstration of spatially-localized events which comprise cardiac SOCE and identify, at least in part, the molecular machinery responsible for these events. Additionally, we identify a high prevalence of SOCE proteins and functional events at the intercalated disk (ID), which electrically and mechanically couples cardiac myocytes. Last but not least, our data suggest a novel arrhythmia mechanism in CPVT driven by pathological remodeling of SOCE machinery, and consequent enhancement of SOCE. Taken together, these results provide much- needed insights into the nature and roles of SOCE in the normal and diseased heart, and as a potential target for anti-arrhythmia therapy.

## Results

We examined the compartmentalization of SOCE in ventricular myocytes derived from WT mice and mice affected by catecholaminergic polymorphic ventricular tachycardia (CPVT) using a combination of functional live cell (2D resonant-scanning confocal Ca^2+^ imaging) and molecular visualization (confocal immunofluorescence microscopy and STORM). These studies revealed that in cardiomyocytes functional SOCE sites and the corresponding molecular complexes of STIM1-ORAI1 are enriched at discrete regions at the intercalated discs (IDs). Furthermore, redistribution of STIM1 and ORAI1 from interior regions to the IDs resulted in augmented SOCE in myocytes from arrhythmia-prone (CPVT) hearts.

### Cardiac SOCE observed as **Lo**cal **C**a^2+^**e**ntry (LoCE) signals

**Figure 1:**
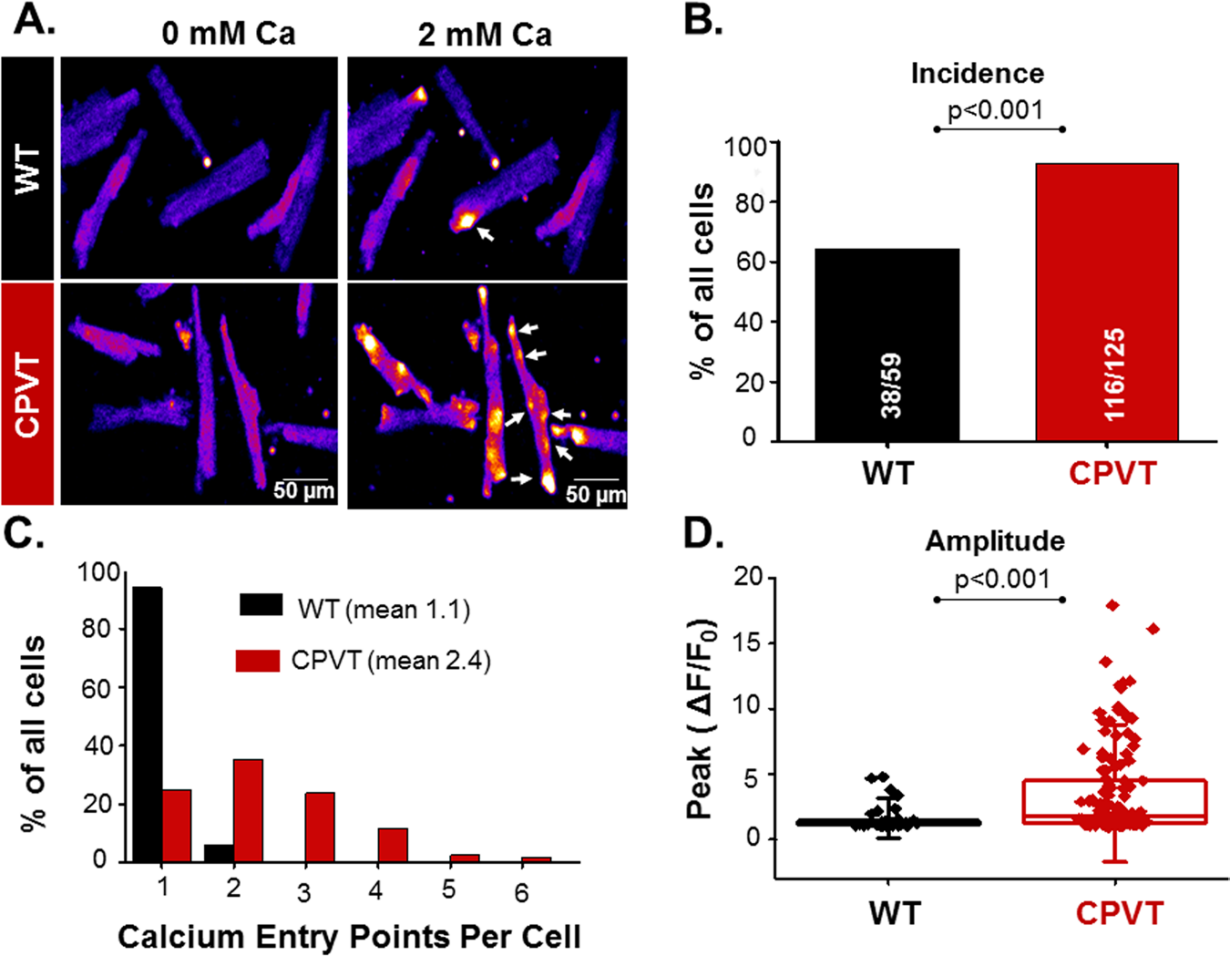
SOCE visualization in WT and CPVT ventricular myocytes. (**A**) Resonant-scan imaging of SR Ca^2+^-depleted WT and CPVT myocytes reveals LoCEs (arrows) upon increasing [Ca^2+^]o from 0 (left) to 2 mM (right). Scale bars are 50 µm. Myocyte SR Ca^2+^ was depleted by 1 µM TG added to a Ca^2+^-free bathing solution. (**B**,**C**) Graphs of the fraction of WT and CPVT cells exhibiting LoCEs and one or multiple (2–6) LoCEs per myocyte, respectively. (**D**) Peak fluorescence intensity of LoCEs recorded in WT and CPVT myocytes. Data presented as mean ± SE from 184 myocytes from 16 animals.

To assess cardiac SOCE and its changes in proarrhythmic cardiac disease, we performed 2D resonant-scanning confocal Ca^2+^ imaging in Fluo-4 AM-loaded WT and CPVT myocytes. SOCE measurements we performed using a standard protocol that consisted of removal of extracellular Ca^2+^ ([Ca^2+^]_O_), and application of the SERCA inhibitor thapsigargin (2 µM) to deplete the SR Ca^2+^ stores, followed by a rapid restoration of [Ca^2+^]_O_ (2 mM). Potential contributions to the Ca^2+^ signal of the L-type Ca^2+^ channels and NCX were minimized with their respective inhibitors verapamil (10 µM) and SEA0400 (1 µM)^14,15,20^. Notably, upon reintroduction of extracellular Ca^2+^, the SR Ca^2+^-depleted myocytes exhibited transient increases in background fluorescence in discrete cellular regions (Fig. 1A). No such local Ca^2+^ elevations were observed in myocytes subjected to extracellular Ca^2+^ withdrawal-reintroduction without SR Ca^2+^ depletion (Supplemental Fig. 1). The local Ca^2+^ elevations observed on restoration of [Ca^2+^]_O_ in the SR Ca^2+^-depleted myocytes are consistent with Ca^2+^ entry through SOC channels in SOCE microdomains. We dubbed these local Ca^2+^ entry signals LoCEs.

### LoCEs are upregulated in CPVT myocytes

**Figure 2:**
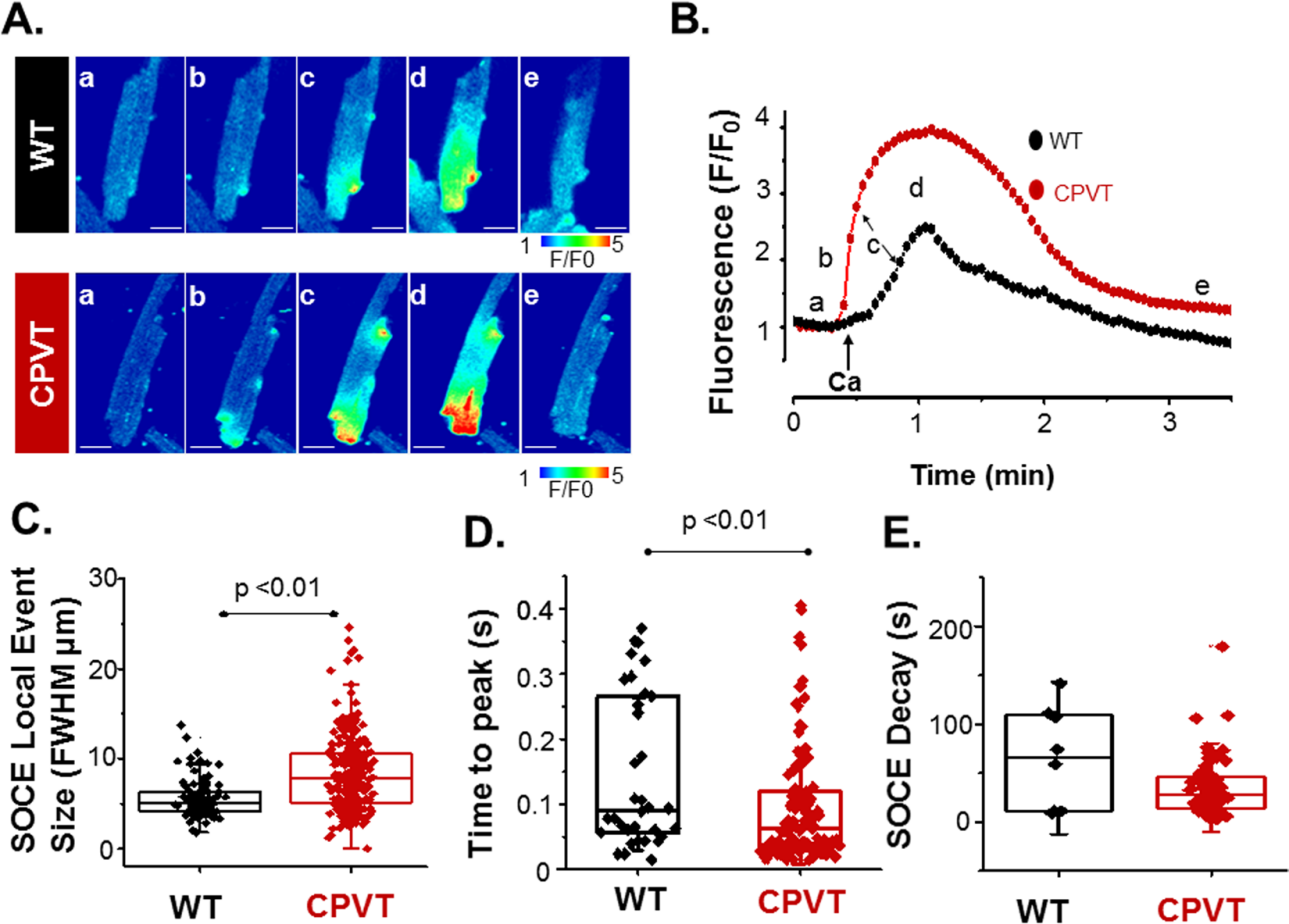
Spatio-temporal properties of LoCEs in WT and CPVT myocytes. (**A**) Representative time-laps images of LoCEs in SR Ca^2+^-depleted WT and CPVT myocytes upon reintroduction of 2 mM extracellular Ca^2+^. Scale bars are 10 µm. Time points of (**B**). Time-dependent peak fluorescence profiles of LoCEs from Panel A for WT (black line) and CPVT (red Line) myocytes. Low case letters (a–e) denote the timing of the corresponding images in Panel A for both WT and CPVT myocytes. (**C**–**E**) Plots of average local fluorescence signal width at half-maximum amplitude (FWHM), time to peak and decay for LoCEs, respectively, in WT and CPVT myocytes. Data presented as mean ± SE from 57 myocytes from 11 animals.

Next, we compared the prevalence and spatiotemporal characteristics of LoCEs in WT and CPVT myocytes to identify disease-driven alterations. Only approximately 60% of WT myocytes exhibited LoCEs (Fig. 1B), with just one, or two isolated events per cell (Fig. 1A,C). In contrast, in CPVT LoCEs occurred in a vast majority of myocytes (90%); Additionally, LoCE signal intensity and the number of signals per cell were increased CPVT relative to WT myocytes (Fig. 1A–D). Furthermore, CPVT myocytes displayed larger LoCEs (diameter at half maximum amplitude (DHMA), 8 µm vs. 5 µm in control) with shorter time to peak (100 ms, vs. 250 ms in control, p < 0.05), albeit no significant change in decay time, relative to control (Fig. 2). Thus, the Ca^2+^ microdomains detected as LoCEs are markedly upregulated in CPVT myocytes compared to WT.

### LoCEs are suppressed by inhibitors of SOCE and absent in STIM1 KO myocytes

**Figure 3:**
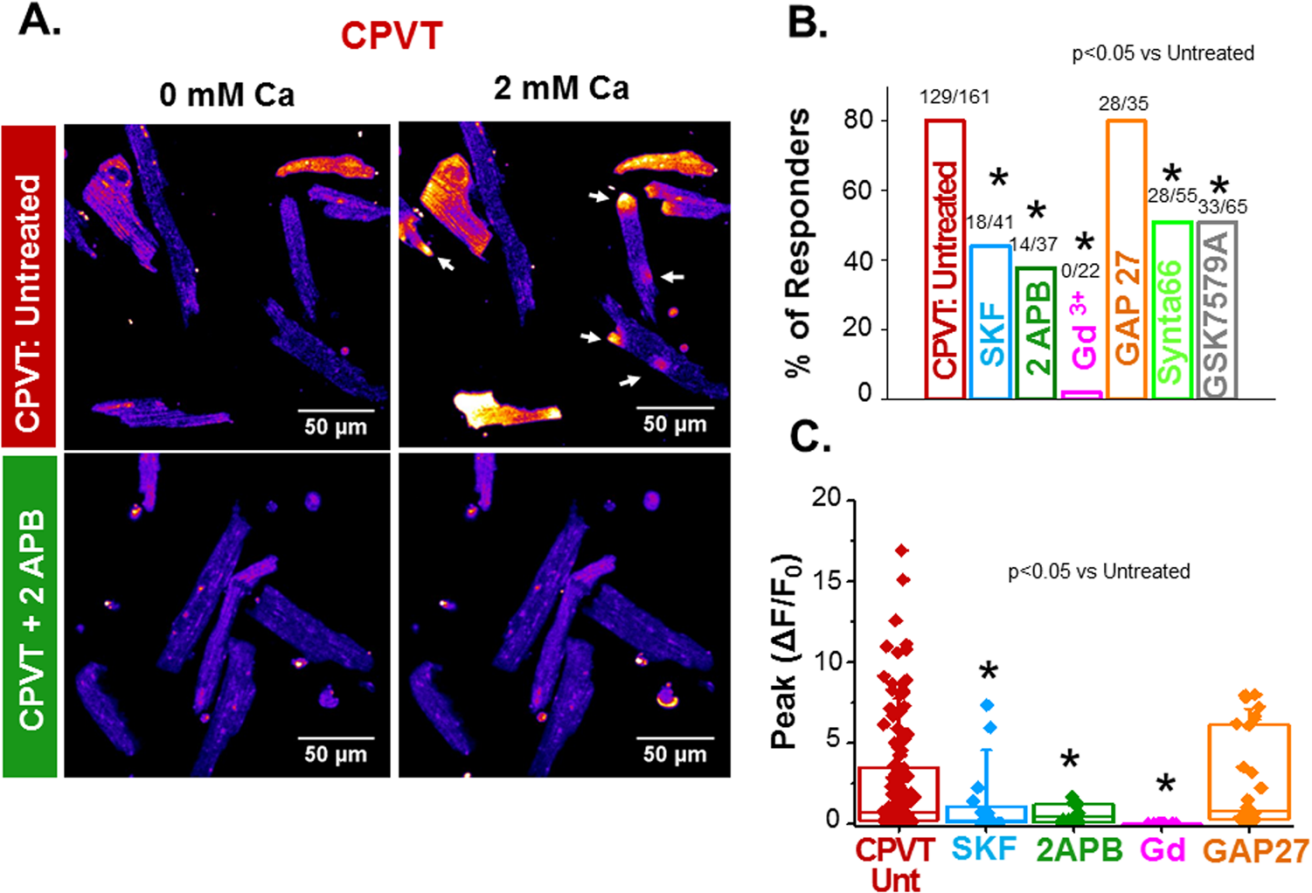
LoCEs are inhibited by SOCE and ORAI inhibitors. (**A**) Representative images of untreated and 2 APB-treated SR Ca^2+^-depleted CPVT myocytes (lower and upper images, respectively) before and after increasing [Ca^2+^]o from 0 to 2 mM (left-and right-hand images, respectively). (**B**,**C**) Summary data on the effect of SOCE inhibitors SKF (10 µM), 2 APB (50 µM) and Gd3+ (10 µM), connexin hemichannel inhibitor, GAP27 (300 µM), ORAI blockers Synta66 (10 µM) and GSK7579A (10 µM) on the fraction of myocytes exhibiting LoCEs and on the peak amplitude of these signals. Data presented as mean ± SE from 416 myocytes from 16 animals.

**Figure 4:**
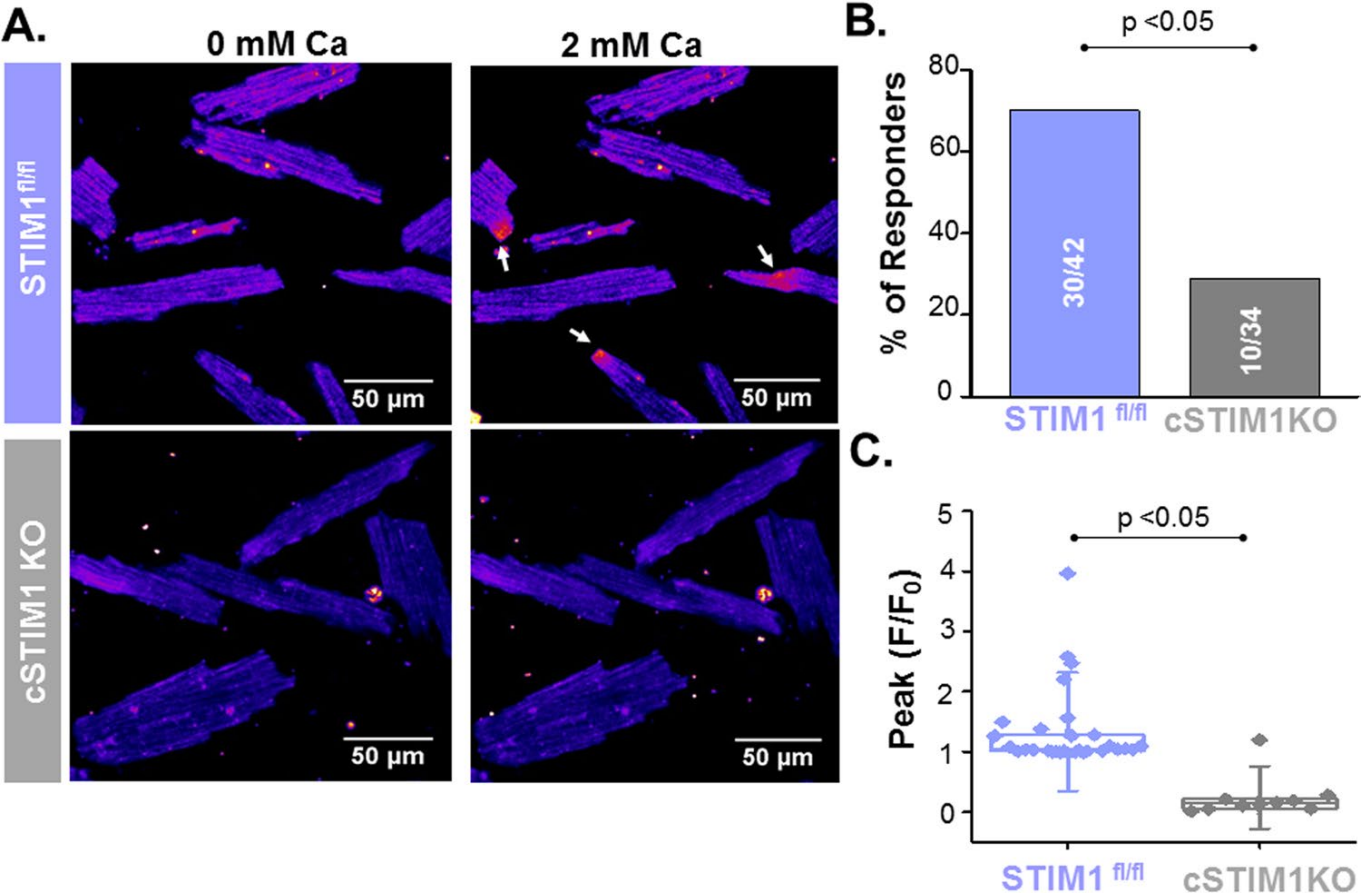
LoCES are abolished in STIM1 KO myocytes. (**A**) Representative images of STIM KO (STIM1fl/fl Cre+) and control (STIM1fl/fl Cre*−*) SR Ca^2+^-depleted myocytes before and after increasing [Ca^2+^]o from 0 to 2 mM (left- and right-hand images, respectively). Scale bars are 50 µm. (**B**,**C**) Summary data on the effect of STIM1 KO on the fraction of myocytes exhibiting local fluorescence elevations and on the peak amplitude of these signals. Data presented as mean ± SE from 76 myocytes from 6 animals.

We further tested the role of SOCE in the observed LoCEs, by pharmacologically inhibiting the former. Nonselective SOCE inhibitors SKF96365 (SKF; 10 µM), 2APB (50 µM) or gadolinium (10 µM), diminished the incidence and amplitude of LoCEs, in CPVT myocytes (Fig. 3). Similar inhibition of LoCEs was attained by the selective ORAI channel inhibitors, Synta66 (10 µM) or GSK7579A (GSK; 10 µM)^21,22^. Given the pivotal role of STIM1 in SOCE^2,8^, we also examined LoCEs in a cardiac specific STIM1 knock-out (c-STIM1KO) mouse. As expected, we were unable to elicit LoCEs in c-STIM1KO myocytes using our standard protocol for measuring these signals, whereas corresponding littermate STIM1-expressing myocytes behaved similar to WT myocytes (Fig. 4). LoCEs were insensitive to selective inhibition of gap junction hemichannels (GAP27; 300 µM) (Fig. 3B,C), suggesting that these channels are unlikely to account for LoCEs formation. These results further support the notion that LoCEs represent the activity of SOCE microdomains in cardiomyocytes. They also implicate ORAI channels as significant determinants of LoCEs in CPVT myocytes.

### SOCE inhibition alleviates arrhythmogenic Ca^2+^ waves in CPVT myocytes

**Figure 5:**
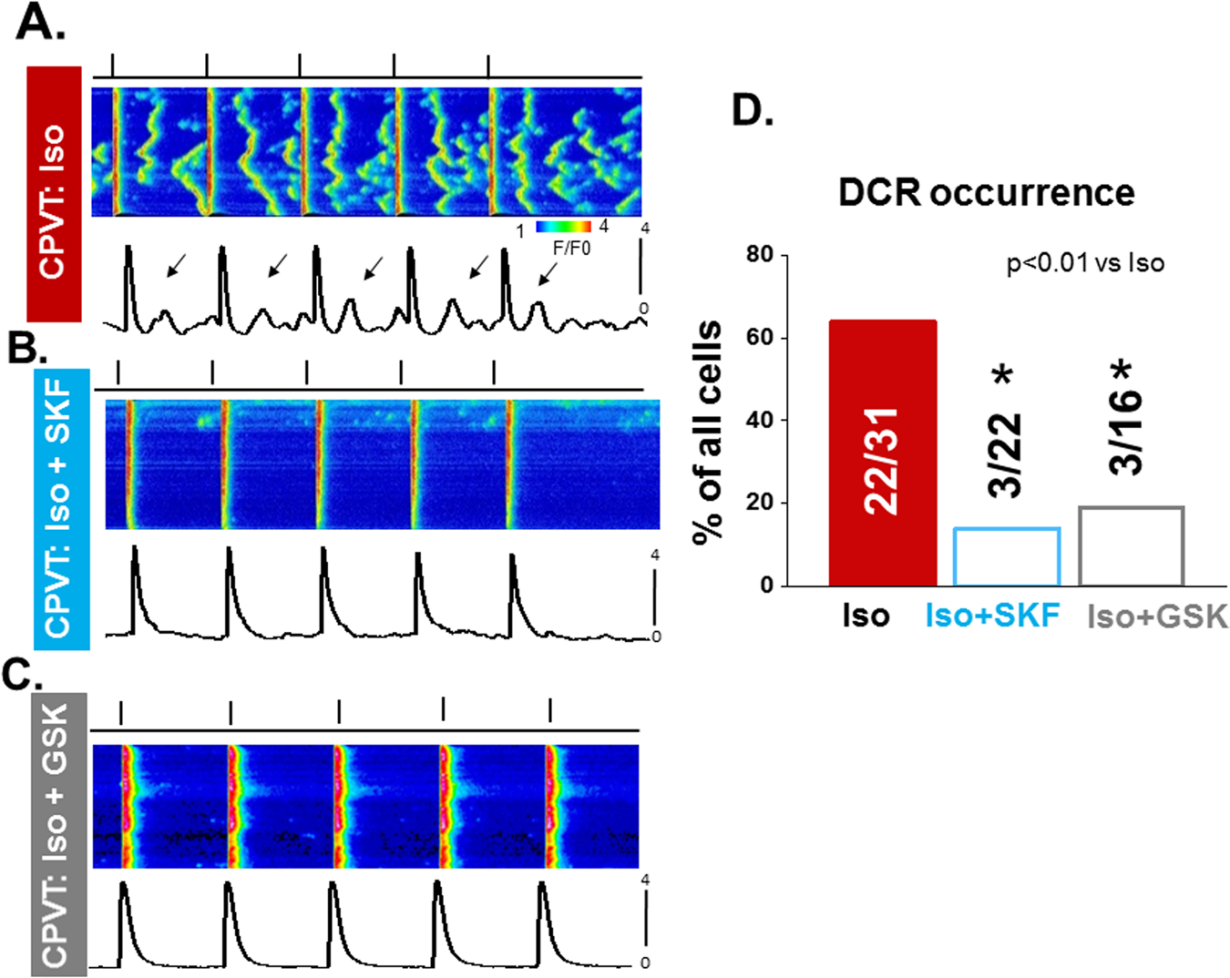
Antiarrhythmic effects of a non-selective SOCE and ORAI selective inhibitors SKF96365 (SKF) and GSK7579A (GSK), respectively. Both SKF and GSK reduced spontaneous Ca^2+^ waves in CPVT myocytes. Line scan images of fluo-3 fluorescence of CPVT myocyte paced at 1 Hz in the presence of 100 nM isoproterenol (Iso) alone (**A**,**B**) in presence of 100 nM Iso plus 10 µM of SKF and C.100 nM Iso plus 10 µM of GSK. (**D**) Diastolic Ca^2+^ wave occurrences in CPVT myocytes recorded with Iso alone, Iso plus SKF and Iso plus GSK. Data presented as percentage of cells exhibiting calcium waves from a total of 5 animals.

In order to probe the physiological relevance of enhanced SOCE in CPVT, we assessed the effect of SOCE inhibition on the propensity for arrhythmogenic Ca^2+^ waves following cholinergic stress (isoproterenol, 100 nM) in CPVT myocytes. Rapid confocal linescan imaging revealed expected spontaneous Ca^2+^ waves occurring in 71% (22/31 cells; Fig. 5) of the CPVT myocytes tested. Notably, SOCE inhibition by the nonselective (SKF, 10 µM) or ORAI-selective (GSK, 10 µM) inhibitors significantly suppressed arrhythmogenic Ca^2+^ waves (13.6% cells and 18.7%, respectively). At the same time, SKF (10 µM) had no significant effects on Ca^2+^ waves in c-STIM1KO myocytes (Supplemental Fig. 5). These results suggest that arrhythmogenic Ca^2+^ waves in cardiac myocytes are associated with STIM1- and ORAI- dependent SOCE and implicate pathologically enhanced SOCE, in the genesis of triggered arrhythmias in CPVT.

### LoCEs occur preferentially at intercalated discs

**Figure 6:**
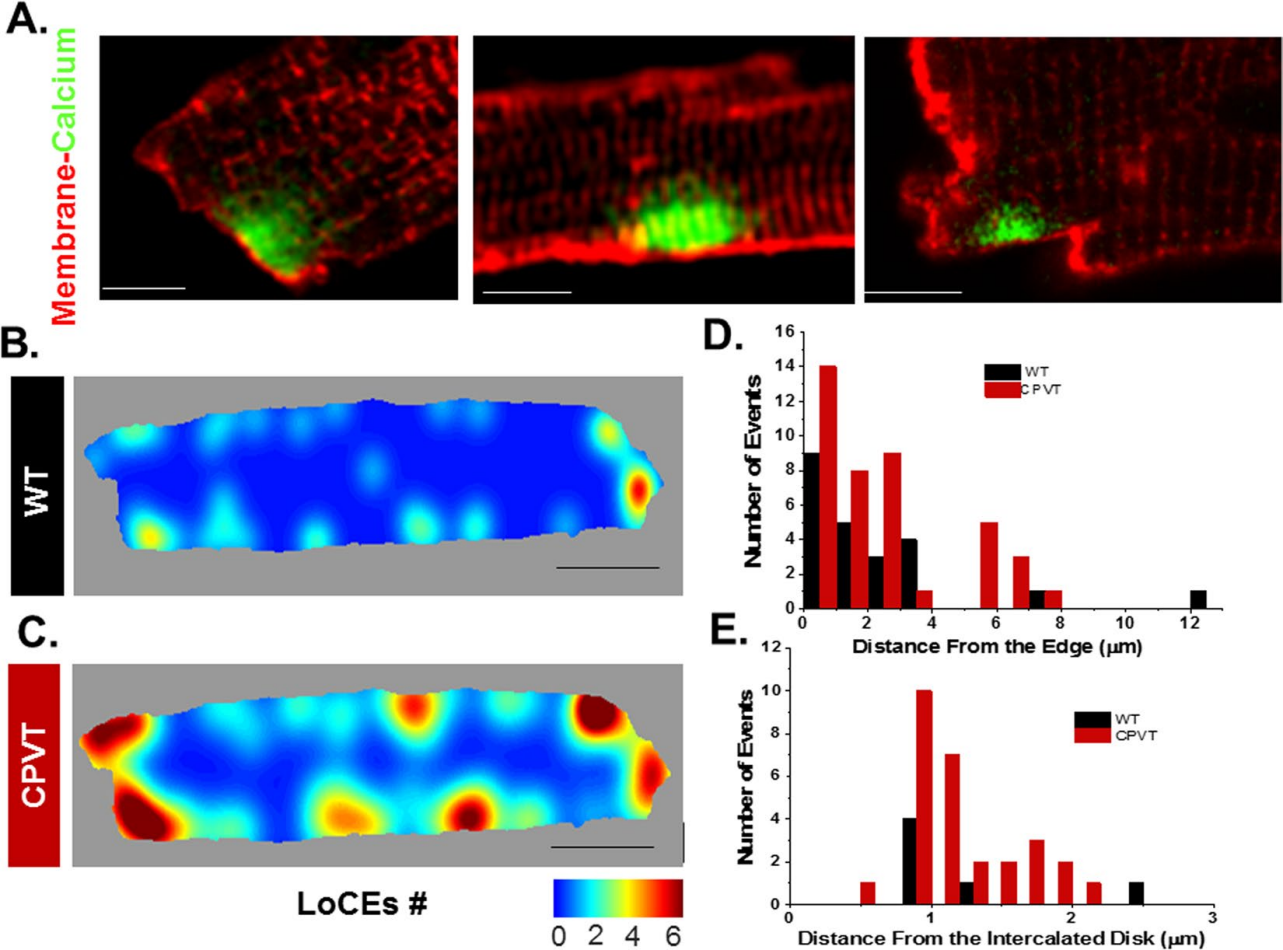
Subcellular localization of LoCEs in WT and CPVT myocytes. (**A**) Representative images of local Ca^2+^ entry (green) in SR Ca^2+^-depleted CPVT myocytes stained with the membrane dye di4-ANNEPs (red). Scale bars are 5 µm. (**B**,**C**) Cumulative localization maps of LoCEs constructed from the original data obtained in WT and CPVT myocytes, respectively. (**D**,**E**) Distributions of distances from LoCE peak location to cell edge along cell periphery or to the ID, respectively. Data presented as mean ± SE from 184 myocytes from 16 animals.

While in skeletal muscle, SOCE has been localized to the T-Tubule-SR junction that houses the machinery for ECC^21,23^, the localization of SOCE in cardiac myocytes is unknown. Therefore, we performed LoCEs imaging with concurrent sarcolemmal labeling (di-4-ANEPPS; Fig. 6A) to assess its localization in WT and CPVT myocytes. These experiments revealed that LoCEs preferentially occur at sites adjacent to surface sarcolemma and intercalated disks (IDs). Heat maps summarizing our experimental observations demonstrate LoCEs occurring predominantly at the myocyte periphery, particularly longitudinal ends, at sites consistent with IDs (Fig. 6B–D). This preferential ID occurrence of LoCEs was enhanced in CPVT myocytes relative to WT. These data suggest that cardiac SOCE may occur at the ID in a spatially distinct sub-compartment from canonical calcium cycling.

### Localization of STIM1 and ORAI1 to IDs

**Figure 7:**
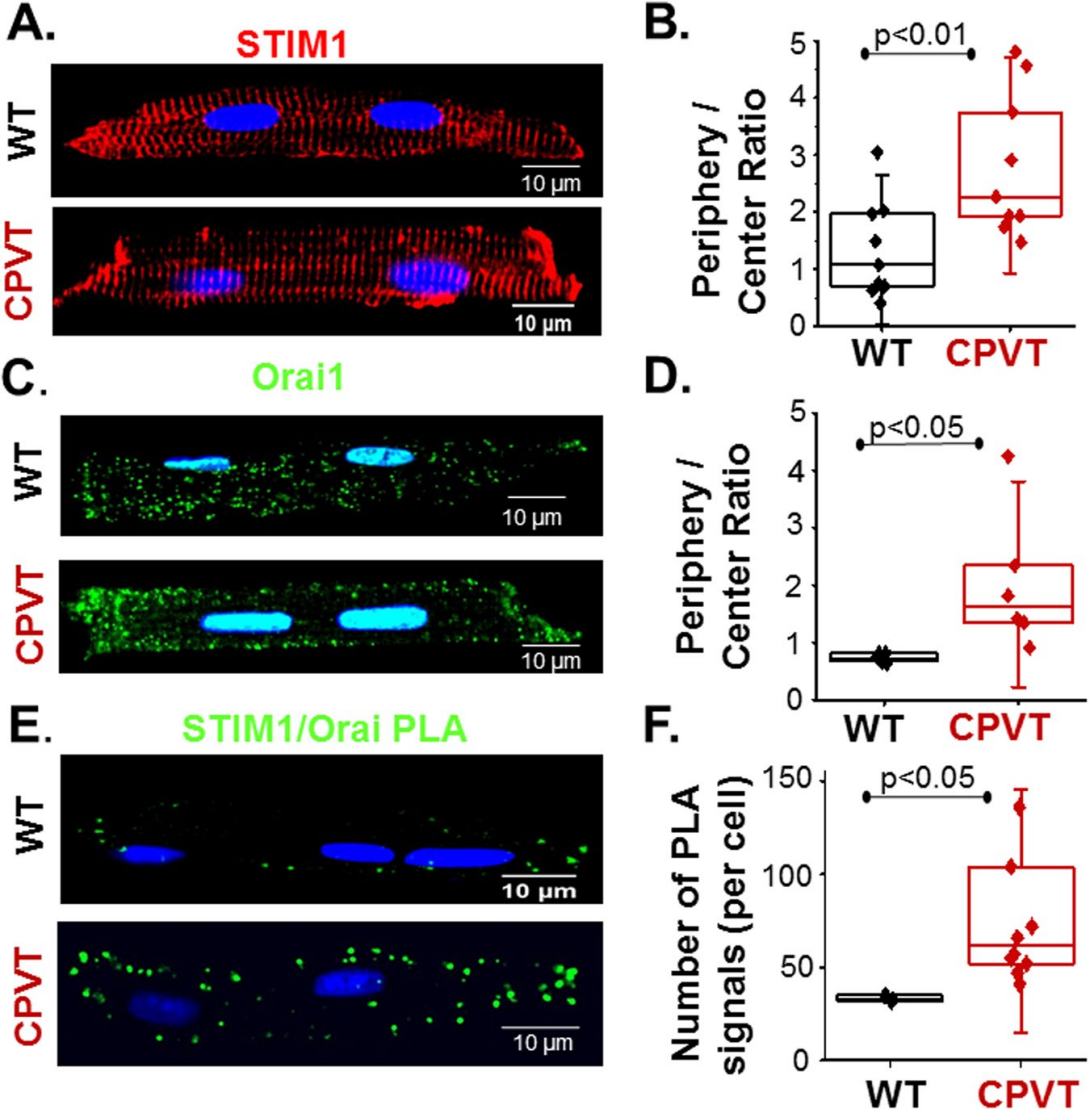
Distribution of STIM1 and ORAI1 and their complexes in WT and CPVT myocytes. (**A**) Representative images of WT (top) and CPVT (bottom) myocytes immunostained for STIM1. Scale bars are 10 µm. (**B**) Summary graph of ratios of myocyte end to - center STIM1 immunofluorescence for WT and CPVT myocytes (as indicated). Region-specific STIM1 immunofluorescence intensities were measured in 4–5 areas at either myocyte ends or interior. (**C**) Representative images of WT (top) and CPVT (bottom) myocytes immunostained for ORAI1. Scale bars are 10 µm. (**D**) Summary graph of ratios of myocyte end to - center ORAI1 immunofluorescence for WT and CPVT myocytes (as indicated). (**E**) Representative images of WT (top) and CPVT (bottom) myocytes subjected to proximity ligation assay (PLA) for STIM1 and ORAI1 co- localization. Scale bars are 10 µm. (**F**) Summary graph of ratios of myocyte end to - center STIM1-ORAI1 PLA signal densities for WT and CPVT myocytes (as indicated). Region-specific immunofluorescence intensities (PLA signal densities) were measured as number of signals per cell. Data presented as mean ± SE from 15 myocytes from 4 animals.

We assessed expression levels and localization of key SOCE constituents, STIM1 and ORAI1, along with several potential SOCE contributors, including STIM2, TRPC 1, 3, 4 and 6, in WT and CPVT myocytes. Based on Western blotting and confocal immunofluorescence analysis, STIM1 and TRPC3 levels were increased in CPVT myocytes, with no difference in the expression of the other proteins (Supplemental Figs 2 and 3). Next the distribution of STIM1 and ORAI1 were examined by immunofluorescence scanning microscopy in WT vs CPVT myocytes. In both cell groups, STIM1 showed a characteristic cross-striated distribution (Fig. 7A). However, STIM1 was markedly enriched at IDs in CPVT myocytes relative to WT. ORAI1 fluorescence demonstrated characteristic speckled distribution throughout WT myocytes (Fig. 7C), with noticeable redistribution to the periphery in CPVT myocytes (Fig. 7D). Further analysis was undertaken by *in situ* proximity ligation assay (PLA), which detects proteins located within 40 nm of each other^24^. PLA corroborated close association of STIM1 and ORAI1 at myocyte periphery including IDs. Consistent with our LoCE imaging (Figs 1 and 6) and immunostaining results (Fig. 7), PLA signal density was markedly enhanced in CPVT relative to WT myocytes. These data provide molecular and structural underpinnings to our functional demonstration of enhanced LoCEs at IDs of CPVT mice.

### Enhanced clustering of STIM1 with Cx43 and N-cadherin in CPVT

**Figure 8:**
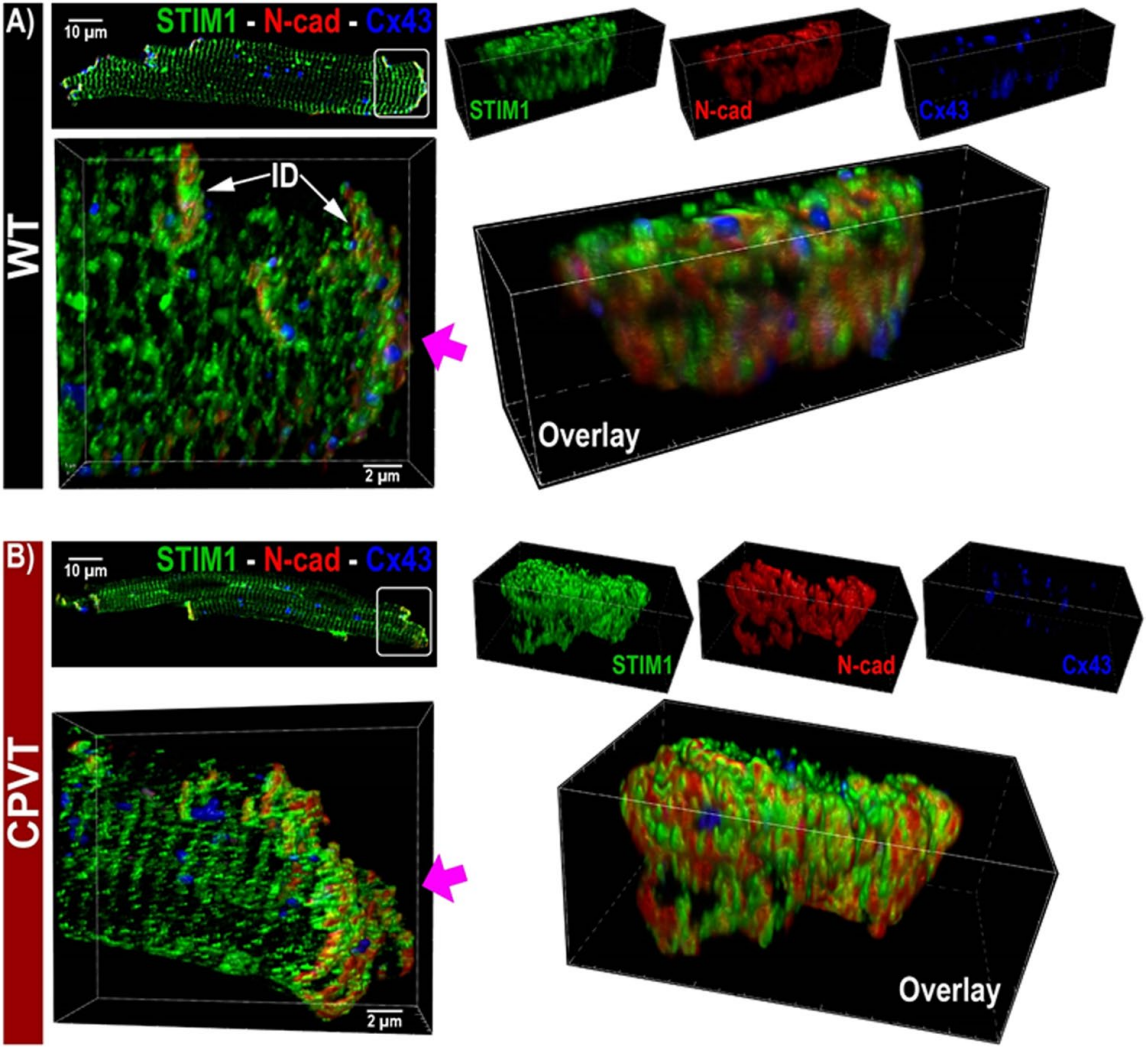
Representative sDCI images of (**A**). WT and (**B**). CPVT murine myocytes illustrate ID enrichment of STIM1. Top left: 2D view of the whole myocyte. Bottom left: 3D view of the region highlighted by the white box in the top left image shows a closer view of the ID. (**C**). High magnification *en face* views of the ID from the bottom left panel. The perspective of this images is indicated by the arrow in the bottom left panel.

Immunohistochemistry combined with sub-diffraction confocal imaging (sDCI; 130 nm lateral and 300 nm axial resolution) was performed to assess the localization of STIM1 relative to other components of the ID such as N-cadherin (N-cad) and connexin 43 (Cx43). In both WT and CPVT murine myocytes, STIM1 was enriched at the ID, along with Cx43 and N-cad (Fig. 8). Closer examination of high magnification views indicated preferential localization of STIM1 to N-cad-rich ID sites over Cx43-rich sites. These data suggest that STIM1 may preferentially localize with N-cad to plicate ID regions.

**Figure 9:**
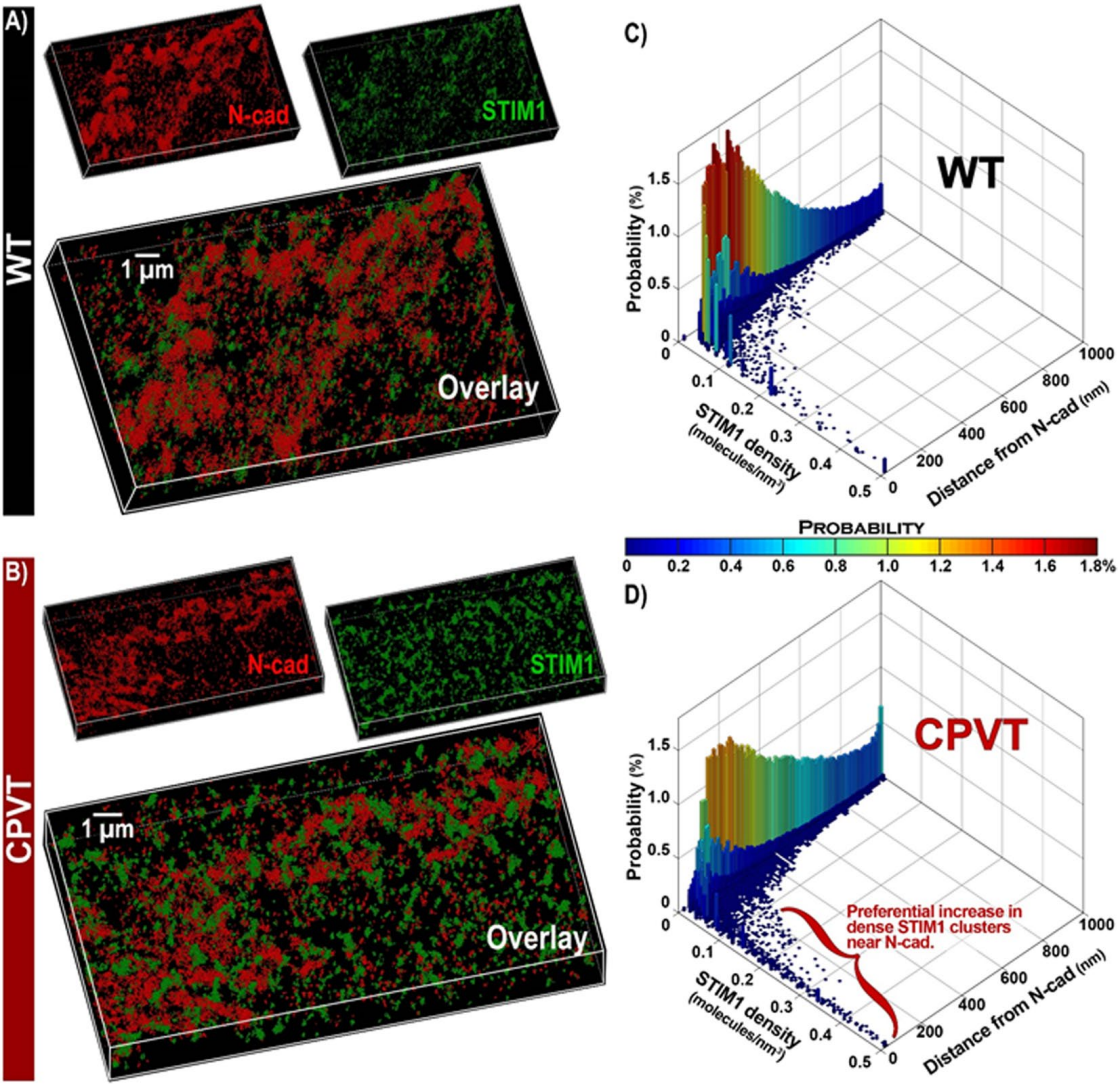
Representative 3D STORM images of *en face* IDs from (**A**). WT and (**B**). CPVT murine hearts labeled for STIM1 and N-cad. Bivariate histograms of STIM1 cluster density and distance from N-cad from (**C**). WT and (**D**). CPVT hearts calculated using the STORM- RLA machine learning approach^10^.

In order to clearly delineate the spatial organization of STIM1 relative to N-cad, we undertook STORM super-resolution microscopy (20 nm lateral and 40 nm axial resolution) in WT and CPVT murine myocardium labeled for N-cad (red) and STIM1 (green). 3D end-on views of IDs show clusters of STIM1 molecules preferentially localized adjacent to N-cad clusters in both WT and CPVT hearts (Figs 8B and 9A,B, respectively). Quantitative analysis of STORM data 50 from WT murine hearts revealed preferential localization of dense STIM1 clusters near N-cad (Fig. 9C) with a median distance of 70 ± 6 nm from ID-localized STIM1 to N-cad. Overall, 69.5 ± 2.3% of STIM1 was located within 100 nm of N-cad, with 10 fold higher STIM1 density within 100 nm of N-cad compared to other ID sites. Quantitative analysis revealed even greater association between STIM1 and N-cad in CPVT hearts relative to WT with a larger population of dense STIM1 clusters located close to (<100 nm) N-cad (Fig. 9D): The median distance between STIM1 and N-cad was reduced to 45 ± 7 nm (p < 0.05 vs WT) and 85.6 ± 3.7% of STIM1 was located within 100 nm of N-cad (p < 0.05 vs WT). These data are consistent with increased association of STIM1 with N-cad in the IDs of CPVT hearts in comparison to WT hearts. The increase in the population of dense, N-cad-adjacent STIM1 clusters is also consistent with the increased magnitude of LoCEs in CPVT myocytes compared to WT (Fig. 2).

## Discussion

In this study, we provide novel insights into the functional properties, and molecular underpinnings of SOCE in the adult cardiac myocyte. Foremost, we identify highly localized, transient Ca^2+^ entry events (LoCEs) that comprise cardiac SOCE. In a previously unanticipated finding, we report that LoCEs and their molecular machinery are concentrated at the myocyte periphery, particularly at the ID, in close proximity to intercellular mechanical junctions. Furthermore, we find that SOCE proteins and LoCEs are upregulated at the ID in the arrhythmogenic Ca^2+^ disease (CPVT). Segregation of SOCE to ID’s, a spatially and functionally distinct compartment from the ECC domain, may facilitate a special role for SOCE in cardiac physiology. Expansion of the SOCE domain in cardiac disease promotes Ca^2+^-dependent arrhythmogenesis. Overall, our findings hold important implications for the understanding of cardiac SOCE and its role in in health and disease.

### LoCEs – represent microdomains of SOCE

Although SOCE is a local process^17,25,26^, it has been commonly studied by measuring averaged myocyte Ca^2+^ levels or whole-cell Ca^2+^ currents. While informative, these studies left unclear the spatial organization of SOCE within the cardiomyocyte and its relation to canonical excitation-contraction coupling (ECC). In this study, we demonstrate, for the first time, local Ca^2+^ entry signals, LoCEs, that represent distinct SOCE microdomains in cardiac muscle. Furthermore, we report that these local Ca^2+^ entry sites were preferentially found at the myocyte periphery, particularly at IDs. The notion that these signals reflect SOCE is supported by the following evidence: (1) LoCEs were observed only under conditions supporting SOCE (i.e. depleted SR Ca^2+^ stores, Supplemental Fig. 1) when alternative pathways of Ca^2+^ entry (I_Ca_, NCX) were inhibited; (2) LoCEs were inhibited by both nonspecific (e.g SKF96365, Gadolinium [Gd^3+^], 2APB) and more ORAI1-selective SOCE inhibitors (e.g. Synta66 and GSK7579A) and diminished by STIM1 KO (Figs 3 and 4), and (3) LoCE occurrence spatially corresponded with sites of STIM1, ORAI1 and TRPC1/4 colocalization (Figs 7 and 8 and Supplemental Fig. 2). Moreover, we also found that both, LoCEs and the SOCE machinery responsible (STIM1 and ORAI1) were enhanced in CPVT myocytes relative to WT (Figs 1 and 2 and Supplemental Fig. 3). These results are consistent with previous reports of compartmentalized SOCE in non-cardiac cell types^17,21,26,27^. Furthermore, CPVT myocytes displayed a high propensity for arrhythmogenic Ca^2+^ waves, which were suppressed by SOCE inhibition (Fig. 5). Taken together, our results represent the first identification of SOCE microdomains in cardiac myocytes, demonstrate SOCE enhancement in CPVT, and implicate SOCE in arrhythmogenesis.

### SOCE microdomains are preferentially localized to ID

Although previous immunostaining studies suggested that STIM1 is localized to Z-lines^27,28^, where SOCE occurs in cardiac myocytes is unknown. While we confirmed this striated pattern of STIM1 distribution, unexpectedly, few SOCE signals were observed at sites corresponding to T-tubules (Fig. 6). Instead, LoCEs predominantly occurred at myocyte ends, near the ID, (Fig. 6). This pattern, noted in WT myocytes, was significantly enhanced in CPVT myocytes, where it was associated with a significant redistribution of STIM1 and ORAI1 to the ID (Figs 1, 6 and 7). The lack of LoCEs at T-tubules suggests that the role of T-tubular STIM1 may extend beyond SOCE to include functions such as regulation of SERCA Ca^2+^ uptake via PLN^28,29^ and/or maintenance of SR structure by STIM1^23,26^. On the other hand, the observed localization of SOCE near intercalated discs implies a function(s) related to these cardiac intercellular structures (see below).

Functional SOCE requires complexation of STIM1 with ORAI1 (and or other SOCE channels such as TRPCs)^2,17,30^. Thus, the enhanced concentration of LoCEs at the IDs of CPVT myocytes could result from either increased expression of SOCE proteins or their enhanced complexation or both. Our results demonstrated that SOCE remodeling in CPVT involves increases in both total and regional STIM1 (Fig. 7 and Supplemental Fig. 3) and enhanced STIM1-ORAI1 complexation at the ID (Fig. 7). Additionally, STORM revealed denser STIM1 clusters in CPVT myocytes (Fig. 9), consistent with increased LoCE magnitude (Fig. 2). Moreover, in non-cardiac cells it has been shown that ORAI1 and TRPC monomers can interact to form SOC channels^31^. Therefore, further research will be needed to fully delineate the roles of other potential SOCE determinants, including different TRPC channel isoforms (e.g. 1, 3, 4, 6) and STIM1 splice variants (e.g. short and long)^32–34^, shown to contribute to SOCE in various cell types. Similarly, interactions of SOCE constituents with different domain-specific modulatory/anchoring proteins (e.g. actin, cadherins)^5,23^ will need to be examined to determine the underlying mechanisms of SOCE remodeling in cardiac disease.

### SOCE microdomains, cardiac hypertrophy and arrhythmogenesis

Although the physiological role and modes of operation of SOCE in cardiac muscle remain to be fully clarified, SOCE has been linked to myocyte development and hypertrophic growth^6,13,35,36^. Several downstream signaling pathways have been suggested to mediate SOCE’s regulatory influence, including calcineurin-nuclear factor of activated T cells (NFAT) and the Akt/protein kinase B (PKB)-mammalian target of rapamycin (mTOR)^2,6,8^. However, how SOCE could provide the required Ca^2+^ signal for such roles, given a backdrop of massive Ca^2+^ fluxes derived from EC coupling, remains unclear. One possible solution could come from the demonstrated here spatial segregation of SOCE between different myocyte compartments.

CPVT is characterized by absence of structural heart disease^12,37^. This may imply that SOCE facilitation is essential, but not sufficient for hypertrophic remodeling. Thus SOCE might be a part of an early stress response not immediately linked to cardiac hypertrophy.

IDs are emerging as versatile signaling hubs involved in mechanical and electrical communication among adjacent cardiomyocytes, mechanosensing as well as cell proliferation and development^38–43^. Thus, segregation to the ID, a spatially distinct compartment from the ECC domain, may confer upon SOCE a privileged role in modulating cardiac myocyte signaling and function. (Put another way, despite wide differences in Ca^2+^ flux levels, SOCE does not have to compete with ECC to influence cardiac physiology). Aside mediating hypertrophic signaling, ID-localized SOCE is strategically poised to modulate ID structure and function by locally altering intracellular and extracellular Ca^2+^ levels. For example, SOCE-derived intracellular Ca^2+^ could modulate cell-to-cell adhesion and electrical coupling through influencing functions of ID-residing proteins such as N-Cadherin, desmoplakin^5,30,44,45^ and or Cx43^40,46–48^. SOCE could also play a role in the maintenance of the ID structure and function via regulation of protein synthesis, trafficking and targeting^3,38,49^.

Ca^2+^ dependent arrhythmias, including CPVT, have been attributed to aberrant Ca^2+^ release via dysregulated RyR2s, resulting in membrane potential disturbances and triggered activity^50–52^. Enhanced SOCE (e.i. increased Ca^2+^ entry) could contribute to arrhythmogenesis through stimulation of RyR2s in areas near the ID^53^. In particular the demonstrated expansion of the SOCE Ca^2+^ domain could contribute to arrhythmogenesis through Ca^2+^ “spillover” from the SOCE pool to the ECC pool, thereby facilitating aberrant SR Ca^2+^ release. In support of this possibility, pharmacological inhibition of SOCE alleviated arrhythmogenic Ca^2+^ release in CPVT myocytes (Fig. 5). Consistent with our results, STIM1 overexpression has been reported to exacerbate arrhythmogenic disturbances in Ca^2+^ handling and membrane potential^6^.

In addition to promoting Ca^2+^-dependent triggered arrhythmia, ID-localized SOCE could also contribute to the arrhythmia substrate through disruption of intercellular mechanical and electrical coupling and conduction slowing^54,55^. Indeed, recent findings identify ID adhesion^55,56^ and conductance^38,40^ as powerful modulators of arrhythmia risk. In this context, the preferential localization of SOCE to N-cad-rich ID sites, demonstrated by STORM (Fig. 9), suggests that SOCE may have a greater impact on mechanical junctions between myocytes compared to gap junctions (C × 43), which localize to distinct parts of the ID from mechanical junctions (N-cad). Furthermore, the preferential localization of STIM1 to N-cad-rich ID sites was significantly elevated in CPVT compared to WT, suggesting that the aforementioned mechanisms may be important in the pathophysiology of cardiac arrhythmias. However, the elucidation of the exact role and specific mechanisms of SOCE participation in cardiac arrhythmogenesis will require further studies.

### Limitations

Our measurements of SOCE/LoCEs were performed under conditions optimized for SOCE detection. Thus, future studies will be required to assess the extent of SOCE activation and consequent Ca^2+^ entry in myocytes under more physiological conditions. Additionally, the role of SOCE in cellular arrhythmia was investigated using pharmacological tools. While useful for initial investigation and analysis, they lack specificity^22,31,57^. Therefore, to avoid this issues, future experiments will use CPVT and CPVT-c-STIM1-KO myocytes to fully assess SOCE role in calcium dependent arrhythmogenic disease. Although revealing roles of STIM1 and ORAI1 in the formation of functional SOCE sites and arrhythmogenesis, the present study has not clearly defined the contribution of other potential SOCE constituents including different isoforms of TRPCs (1, 3, 4, 6). Elucidation of the contribution of these proteins must await further studies.

## Methods

All animal procedures were approved by The Ohio State University Institutional Animal Care and Use Committee and conformed to the Guide for the Care and Use of Laboratory Animals published by the US National Institutes of Health (NIH Publication No. 85-23, revised 2011).

### Mouse models

A total of 30 mixed gender male/female from 12–32 week Calsequestrin 2 (CPVT.R33Q) knock-in mice were used for the present study; 20 age matched mixed gender C57BL/6 mice (Jackson Laboratory) were used as WT controls. Cardiac specific STIM1KO was generated by cross-breeding STIM1^fl/fl^ mice (B6(Cg)-STIM1tm1Rao/J) with Myh6-cre (B6.FVB-Tg(Myh6-cre)2182 Mds/J) from Jackson laboratory. STIM1 KO mice that were STIM1^fl/fl^ *Cre*+ were used as cardiac specific STIM1KO while STIM1^fl/fl^ *Cre−* were used as controls. A total of 6 STIM1 KO were used for the present studies and 6 age matched mixed gender Control (STIM1^fl/fl^ *Cre−*).

### Ventricular cardiomyocyte isolation and LoCES imaging

Intact ventricular myocytes were obtained by enzymatic digestion as previously described^58^. Briefly, mice were anesthetized with isoflurane, hearts were rapidly excised, cannulated through the aorta, and perfused with Ca^2+^ free tyrodes (in mM): 140 NaCl, 5.4 KCl, 0.5 mM MgCl2, 10 HEPES and 5.5 Glucose pH 7.4 at RT using a warm water jacketed gravitational Langendorff apparatus with a set temperature of 32–33 °C. Heart was perfused for 5 minutes with enzyme free Ca^2+^ free Tyrodes in order to clean any blood remnants from the vessels. Enzymatic digestion was achieved by perfusing the heart with low Ca^2+^ (0.2 mM) Tyrodes and liberase TH Research Grade (Roche) as previously described^58^.

Calcium tolerant cells were loaded with Fluo-3/4 AM for 20–30 min in 0.5–1 mM Ca external solution containing: 140 NaCl, 5.4 KCl, 0.5 mM MgCl2, 10 HEPES and 5.5 Glucose pH 7.4 at RT, to monitor intracellular Ca^2+^. Following initial dye loading, to activate LoCES, depletion Solution (0 Ca^2+^ Tyrodes and (in µM): 500 Caffeine, 2 Thapsigargin, 10 Verapamil and 1 SEA 0400) was added to cells, this solution is an optimized version of previously reported depletion solutions^6^ and incubated at room temperature (RT) for10–20 minutes to allow dye de-esterification and cell depletion to occur. Ca^2+^ entry (SOCE) was activated by rapid application of SOCE solution: 2 mM Ca^2+^ Tyrode and (in µM): 2 thapsigargin, 10 verapamil and 1 SEA0400. For studies that included a SOCE blockers (10 µM Gadolinium, 10 µM SKF96365, 50 µM 2APB, 10 µM Synta66 or 10 µM GSK7579A) or the connexin blocker 300 µM GAP27; each blocker was present in both the depletion and SOCE solutions.

Intracellular Ca^2+^ signal was monitored using an Olympus Fluoview 1000 and Nikon A1R confocal microscopes. Fluo-3/4 fluorescence was recorded at (0.41 µm/pixel) with a sampling speed of (8 µs/pixel) for a total of 3 min free run (70 frames at 2.7 sec/frame). Cells were excited using 488 nm argon laser with emission of 500–600 nm using immersion oil 60x objective.

To image LoCEs, cells were kept in depletion solution for the first 20–25 frames (54–60 sec) of the recording. After baseline recording the depletion solution was rapidly removed from the chamber using a disposable transfer pipette while simultaneously SOCE solution was added to the chamber. Recording of LoCES was done as described above.

### Immunofluorescence

Immunofluorescent labeling for confocal^59,60^ and STORM^56^ imaging was performed as before. For cellular imaging studies, freshly isolated cardiomyocytes were placed on laminin coated coverslips and fixed with 2% paraformaldehyde (2 minutes at room temperature: RT). For tissue imaging studies, fresh frozen murine myocardium was cryosectioned (5 µM sections) and fixed with 2% paraformaldehyde (5 minutes at RT) as previously described^60^. Fixed samples were washed with PBS (3 × 10 minutes at RT) followed by blocking/permeabilization (3% fetal bovine serum+ 0.2% triton in PBS; 45 minutes at RT). Samples were then incubated with primary antibody (in PBS+ 10% BSA) overnight at 4 °C. Following primary antibody incubation, samples were washed with PBS (5 × 5 minutes at RT), incubated with fluorescently-labeled secondary antibodies (goat anti- mouse and goat anti-rabbit) for 1 hr at RT and washed again with PBS (3 × 5 minutes at RT). For three color labeling, samples were first labeled with two primary antibodies (anti- STIM1 and anti-Cx43) and corresponding secondary antibodies (conjugated respectively to Alexa Fluor 488 and Alexa Fluor 647) as described above. Subsequently, samples were labeled (overnight at 4 °C) with Ms anti-Ncad (BD biosciences 610921) antibody directly conjugated with Alexa Fluor 568 (Invitrogen Zenon Labeling Kit Z25006) and washed with PBS (5 × 5 minutes at RT).

For confocal microscopy and sDCI, samples were mounted (Invitrogen Prolong Gold with DAPI), and cured (48 hours in the dark at RT) prior to imaging performed using an A1R- HD laser-scanning confocal microscope (Nikon). sDCI was performed using an A1R-HD confocal microscope (Nikon) using a pinhole of 0.4 Airy units with spatial oversampling (pixel size of optical resolution/4.6, z-step size of optical sectioning/4), and 3D deconvolution (NIS Elements software; Nikon). For STORM, samples were optically cleared (Scale U2 buffer) for 48 hours at 4 °C prior to imaging using a Vutara-352 STORM system (Bruker).

Primary antibodies used were: Rabbit anti-STIM1(N-Terminal) (Sigma S6072; 1:1000), Ms anti-ORAI1 (Alomone ALM-025; 1:500), Ms anti–NCad (BD biosciences 610921; 1:2000), Ms anti-C × 43 (Millipore MAB3067; 1:1000) and Ms anti-RyR2 (Invitrogen MA3- 916 1:1,000). For confocal microscopy secondary antibodies were applied at 1:1000 dilution. Images were processed using ImageJ. For STORM, samples were labeled with secondary antibodies conjugated to Alexa Fluor 647 (Invitrogen) and CF 568 (Biotium) and imaged as previously described using a Vutara-352 STORM system (Bruker).

### Ca^2+^ transients imaging

Cell were plated in laminin coated coverslips and loaded with the calcium sensitive dye Fluo-3 or Fluo-4 AM for 20–25 minutes at RT. Dye loading was followed by dye desertification for 20–25 minutes. The fluorescent probe was excited with the 488-nm line of an argon laser and emission was collected at 500–600 nm. Fluo-3/4 fluorescence was recorded in the line scan mode of the confocal microscope (0.414 μm per pixel, 2–5 ms per line) using Olympus FV-1000 confocal microscopy. Cells were constantly perfused with Tyrode solution +100 nM isoproterenol to induce spontaneous diastolic calcium release (DCR). The SOCE blocker SKF96365 (10 µM) or GSK7579A (10 µM) was added to the solution to assess its effect in DCR. Myocytes were paced using at 1 Hz using external platinum electrodes. Any spontaneous diastolic Ca^2+^ release (DCR) event (i.e., wave, wavelet) that increased cell-wide fluorescence intensity above 10% of the signal generated by the preceding stimulated Ca^2+^ transient was included in the analysis. The fluorescence emitted was expressed as F/F0, where F is the fluorescence at time t and F_0_ represents the background signal. All experiments were performed at room temperature (26 °C).

### Proximity ligation assay (PLA)

Freshly isolated cardiomyocytes were placed on laminin coated coverslips and fixed with 2% formalin for 2 minutes. Cells were washed with PBS three times followed by a blocking/permeabilization step (3% fetal bovine serum +0.2% triton in PBS) for a total of 45 minutes. After permeabilization was achieved, cells were washed three times with PBS and incubated with 10% BSA with primary antibody overnight in the fridge. The next day cells were washed 5 times with PBS and the PLA reactions were carried out using appropriate Duolink secondary antibodies (Sigma, St. Louis, Missouri).The same antibodies used for immunofluorescence assays where used for PLA. STIM1 antibody was used at a concentration of 1:500 and ORAI1 1:500.Samples that were incubated with only one of the two primary antibodies before PLA procedure lacked signal. (Data not shown)

### Data analysis

Analysis of SOCE: A threshold for SOCE detection was calculated as an average of mean +3*standard deviations (SDs) of myocyte fluorescence recorded under the SR Ca^2+^ depleted conditions (before application of ‘SOCE solution’). Image pixels exceeding the threshold in response to application of ‘SOCE solution’ were counted and expressed as a percentage of myocyte area. If percentage of these pixels exceeded 1% of myocyte area, the myocyte was considered to exhibit SOCE response. Properties of the local increase in fluorescence (number, peak, width at half-magnitude, location of the peak relative to the cell edge and lateral end) were analyzed following normalization of corresponding image frame to the averaged background image recorded before application of ‘SOCE solution’.

Statistical analysis was completed using Origin and/or Microsoft Excel. Unpaired one tailed student t-test or ANOVA with Fisher test as post hoc test were used to assess statistical significance. For paired data we utilized student t-test. Presence or absence of events such as SOCE or DCR were analyzed using a two tailed Fishers exact test. Outlier data points were excluded by using the outlier calculator in Graph pad with significance level of Alpha 0.05. Only cells that had responses where included in the analysis for dynamics and kinetics of the SOCE signals. Categorical data was analyzed using two tailed Fisher’s exact test in Graph Pad. A p < 0.05 was considered statistically significant.

## Supplementary information

Enhancement of Cardiac Store Operated Calcium Entry (SOCE) within Novel Intercalated Disk Microdomains in Arrhythmic Disease

## Data Availability

All data generated and analyzed during the present study are available upon request from the corresponding author upon reasonable request.

## References

[1] Bagur R, Hajnoczky G (2017). Intracellular Ca(2+) Sensing: Its Role in Calcium Homeostasis and Signaling. Mol. Cell.

[2] Berna-Erro A, Redondo PC, Rosado JA (2012). Store-operated Ca(2+) entry. Adv. Exp. Med. Biol..

[3] Oh-hora M (2008). Dual functions for the endoplasmic reticulum calcium sensors STIM1 and STIM2 in T cell activation and tolerance. Nat. Immunol..

[4] Prakriya M, Lewis RS (2006). Regulation of CRAC channel activity by recruitment of silent channels to a high open-probability gating mode. J. Gen. Physiol.

[5] Soni D (2017). Pyk2 phosphorylation of VE-PTP downstream of STIM1-induced Ca(2+) entry regulates disassembly of adherens junctions. Am. J. Physiol Lung Cell Mol. Physiol.

[6] Correll RN (2015). STIM1 elevation in the heart results in aberrant Ca(2)(+) handling and cardiomyopathy. J. Mol. Cell Cardiol..

[7] Jessica S (2018). Ca(2+) handling remodeling and STIM1L/Orai1/TRPC1/TRPC4 upregulation in monocrotaline-induced right ventricular hypertrophy. J. Mol. Cell Cardiol..

[8] Luo X (2012). STIM1-dependent store-operated Ca(2)(+) entry is required for pathological cardiac hypertrophy. J. Mol. Cell Cardiol..

[9] Ross GR (2017). Enhanced store-operated Ca(2+) influx and ORAI1 expression in ventricular fibroblasts from human failing heart. Biol. Open..

[10] Veeraraghavan R, Gourdie RG (2016). Stochastic optical reconstruction microscopy-based relative localization analysis (STORM-RLA) for quantitative nanoscale assessment of spatial protein organization. Mol. Biol. Cell.

[11] Zhang H (2015). STIM1-Ca2+ signaling modulates automaticity of the mouse sinoatrial node. Proc. Natl. Acad. Sci. USA.

[12] Valle G (2014). Post-natal heart adaptation in a knock-in mouse model of calsequestrin 2-linked recessive catecholaminergic polymorphic ventricular tachycardia. Exp. Cell Res..

[13] Voelkers M (2010). Orai1 and Stim1 regulate normal and hypertrophic growth in cardiomyocytes. J. Mol. Cell Cardiol..

[14] Alansary D (2014). Measuring endogenous ICRAC and ORAI currents with the patch-clamp technique. Cold Spring Harb. Protoc..

[15] Hogan PG, Rao A (2007). Dissecting ICRAC, a store-operated calcium current. Trends Biochem. Sci..

[16] Shim AH, Tirado-Lee L, Prakriya M (2015). Structural and functional mechanisms of CRAC channel regulation. J. Mol. Biol..

[17] Ambudkar IS, de Souza LB, Ong HL (2017). TRPC1, Orai1, and STIM1 in SOCE: Friends in tight spaces. Cell Calcium.

[18] Ong HL, de Souza LB, Ambudkar IS (2016). Role of TRPC Channels in Store-Operated Calcium Entry. Adv. Exp. Med. Biol..

[19] Vaca L (2010). SOCIC: the store-operated calcium influx complex. Cell Calcium.

[20] Eisner DA (2017). Calcium and Excitation-Contraction Coupling in the Heart. Circ. Res..

[21] Boncompagni S (2017). Exercise-dependent formation of new junctions that promote STIM1-Orai1 assembly in skeletal muscle. Sci. Rep..

[22] Singh A (2010). The transient receptor potential channel antagonist SKF96365 is a potent blocker of low-voltage-activated T-type calcium channels. Br. J. Pharmacol..

[23] Darbellay B (2011). STIM1L is a new actin-binding splice variant involved in fast repetitive Ca2+ release. J. Cell Biol..

[24] Radwanski PB (2015). Neuronal Na+ channel blockade suppresses arrhythmogenic diastolic Ca2+ release. Cardiovasc. Res..

[25] Huser J (1999). Focal agonist stimulation results in spatially restricted Ca2+ release and capacitative Ca2+ entry in bovine vascular endothelial cells. J. Physiol.

[26] Parks C (2016). STIM1-dependent Ca(2+) microdomains are required for myofilament remodeling and signaling in the heart. Sci. Rep..

[27] Rosenberg P, Katz D, Bryson V (2019). SOCE and STIM1 signaling in the heart: Timing and location matter. Cell Calcium.

[28] Zhao G (2015). STIM1 enhances SR Ca2+ content through binding phospholamban in rat ventricular myocytes. Proc. Natl. Acad. Sci. USA.

[29] Jousset H, Frieden M, Demaurex N (2007). STIM1 knockdown reveals that store-operated Ca2+ channels located close to sarco/endoplasmic Ca2+ ATPases (SERCA) pumps silently refill the endoplasmic reticulum. J. Biol. Chem..

[30] Cheng KT (2011). Local Ca(2)+ entry via Orai1 regulates plasma membrane recruitment of TRPC1 and controls cytosolic Ca(2)+ signals required for specific cell functions. PLoS. Biol..

[31] Molnar T (2016). Store-Operated Calcium Entry in Muller Glia Is Controlled by Synergistic Activation of TRPC and Orai Channels. J. Neurosci..

[32] McNally BA (2013). The C- and N-terminal STIM1 binding sites on Orai1 are required for both trapping and gating CRAC channels. J. Physiol.

[33] Prakriya M (2013). Store-operated Orai channels: structure and function. Curr. Top. Membr..

[34] Sauc S (2015). STIM1L traps and gates Orai1 channels without remodeling the cortical ER. J. Cell Sci..

[35] Hulot JS (2011). Critical role for stromal interaction molecule 1 in cardiac hypertrophy. Circulation.

[36] Troupes CD (2017). Role of STIM1 (Stromal Interaction Molecule 1) in Hypertrophy-Related Contractile Dysfunction. Circ. Res..

[37] Haugaa KH (2010). High prevalence of exercise-induced arrhythmias in catecholaminergic polymorphic ventricular tachycardia mutation-positive family members diagnosed by cascade genetic screening. Europace..

[38] Lang D (2017). Calcium-Dependent Arrhythmogenic Foci Created by Weakly Coupled Myocytes in the Failing Heart. Circ. Res..

[39] Leybaert L (2017). Connexins in Cardiovascular and Neurovascular Health and Disease: Pharmacological Implications. Pharmacol. Rev..

[40] Li J, Patel VV, Radice GL (2006). Dysregulation of cell adhesion proteins and cardiac arrhythmogenesis. Clin. Med. Res..

[41] Liu MB (2015). Delayed afterdepolarizations generate both triggers and a vulnerable substrate promoting reentry in cardiac tissue. Heart Rhythm..

[42] Peters NS (1996). New insights into myocardial arrhythmogenesis: distribution of gap-junctional coupling in normal, ischaemic and hypertrophied human hearts. Clin. Sci. (Lond).

[43] Peters NS (1995). Cardiac arrhythmogenesis and the gap junction. J. Mol. Cell Cardiol..

[44] Antigny F (2011). Thapsigargin activates Ca(2)+ entry both by store-dependent, STIM1/Orai1-mediated, and store-independent, TRPC3/PLC/PKC-mediated pathways in human endothelial cells. Cell Calcium.

[45] Hobbs RP (2011). The calcium ATPase SERCA2 regulates desmoplakin dynamics and intercellular adhesive strength through modulation of PKC&alpha; signaling. FASEB J..

[46] Huang RY (2011). Identification of CaMKII phosphorylation sites in Connexin43 by high-resolution mass spectrometry. J. Proteome. Res..

[47] Jabr RI (2016). Regulation of gap junction conductance by calcineurin through C×43 phosphorylation: implications for action potential conduction. Pflugers Arch..

[48] Xu Q (2012). Gating of connexin 43 gap junctions by a cytoplasmic loop calmodulin binding domain. Am. J. Physiol Cell Physiol.

[49] Somasundaram A (2014). Store-operated CRAC channels regulate gene expression and proliferation in neural progenitor cells. J. Neurosci..

[50] Clusin WT (2003). Calcium and cardiac arrhythmias: DADs, EADs, and alternans. Crit Rev. Clin. Lab Sci..

[51] Lou Q (2015). Alternating membrane potential/calcium interplay underlies repetitive focal activity in a genetic model of calcium-dependent atrial arrhythmias. J. Physiol.

[52] Singh VP (2013). Abnormal calcium cycling and cardiac arrhythmias associated with the human Ser96Ala genetic variant of histidine-rich calcium-binding protein. J. Am. Heart Assoc..

[53] Cerrone M (2017). Plakophilin-2 is required for transcription of genes that control calcium cycling and cardiac rhythm. Nat. Commun..

[54] George SA (2016). Extracellular sodium dependence of the conduction velocity-calcium relationship: evidence of ephaptic self-attenuation. Am. J. Physiol Heart Circ. Physiol.

[55] George, S. A. *et al*. Modulating Cardiac Conduction during Metabolic Ischemia with Perfusate Sodium and Calcium in Guinea Pig Hearts, *Am. J. Physiol Heart Circ. Physiol* (2019). 10.1152/ajpheart.00083.2018 PMC6483020 30707595

[56] Veeraraghavan, R. *et al*. The adhesion function of the sodium channel beta subunit (beta1) contributes to cardiac action potential propagation, *Elife*. **7** (2018). 10.7554/eLife.37610 PMC6122953 30106376

[57] Tanahashi Y (2016). Inhibitory effects of SKF96365 on the activities of K(+) channels in mouse small intestinal smooth muscle cells. J. Vet. Med. Sci..

[58] Ho HT (2016). Muscarinic Stimulation Facilitates Sarcoplasmic Reticulum Ca Release by Modulating Ryanodine Receptor 2 Phosphorylation Through Protein Kinase G and Ca/Calmodulin-Dependent Protein Kinase II. Hypertension.

[59] Radwanski PB (2016). Neuronal Na(+) Channels Are Integral Components of Pro-arrhythmic Na(+)/Ca(2+) Signaling Nanodomain That Promotes Cardiac Arrhythmias During beta-adrenergic Stimulation. JACC. Basic Transl. Sci..

[60] Veeraraghavan R, Radwanski PB (2018). Sodium channel clusters: harmonizing the cardiac conduction orchestra. J. Physiol.

