# Supplementary material for: Enhancement of Cardiac Store Operated Calcium Entry (SOCE) within Novel Intercalated Disk Microdomains in Arrhythmic Disease
Source: Sci Rep. 2019 Jul 15;9:10179. doi: 10.1038/s41598-019-46427-x (PMC6629850; doi:10.1038/s41598-019-46427-x)
Supplement: Supplementary file 1 — Enhancement of Cardiac Store Operated Calcium Entry (SOCE) within Novel Intercalated Disk Microdomains in Arrhythmic Disease [file 41598_2019_46427_MOESM1_ESM.pdf]

Enhancement of Cardiac Store Operated Calcium Entry (SOCE) within Novel Intercalated Disk Microdomains in Arrhythmic Disease

Ingrid M. Bonilla[1,2,4], Andriy Belevych[1,2], Stephen Baine[4], Andrei Stepanov[9], Louisa Mezache[1,5],Tom Bodnar[1,2], Bin Liu[6], Pompeo Volpe[7], Silvia Priori[8], Noah Weisleder[1,2], Galina Sakuta[9], Cynthia A. Carnes[1,3,4], Przemysław B. Radwański[1-4], Rengasayee Veeraraghavan[1,2,5]*, Sandor Gyorke[1,2]*

Affiliations:

[1] Dorothy M. Davis Heart and Lung Research Institute, College of Medicine, The Ohio State University Wexner Medical Center, Columbus, OH, USA

[2] Department of Physiology and Cell Biology, College of Medicine, The Ohio State University, Columbus, OH, US

[3] Division of Pharmacy Practice and Sciences, College of Pharmacy, The Ohio State University, Columbus, OH, US

[4] Division of Pharmacology, College of Pharmacy, The Ohio State University, Columbus, OH, US

[5] Department of Biomedical Engineering, College of Engineering, The Ohio State University, Columbus, OH, US

[6] Department of Biological Sciences, Mississippi State University, Mississippi State, MS, US

[7] Department of Biomedical Sciences, University of Padova, Padova, Italy

[8] Department of Molecular Medicine, University of Pavia, Pavia, Italy

[9] Laboratory of Cell Pathology, Institute RAS, Saint Petersburg, Russia

Supplemental Figure 1.

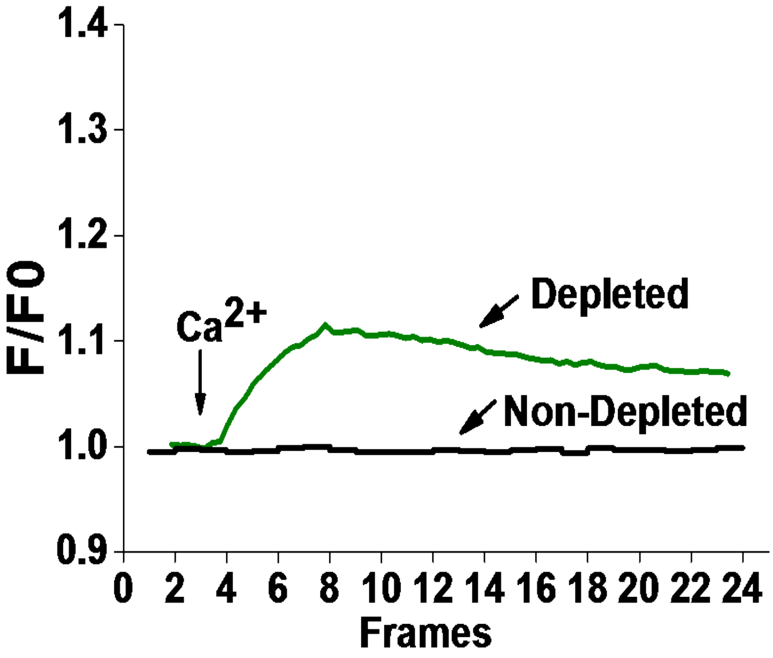

Supplemental Figure 1. LoCEs depend on SR Ca^{2+} store depletion. Representative cell-averaged Fluo-4 fluorescence traces in CPVT myocytes with depleted (green line) and intact (black line) SR Ca^{2+} stores. To activate SOCE myocyte SR Ca^{2+} stores were depleted by application of caffeine (0.5 mM) and thapsigargin (10 μM). SOCE was measured upon reintroduction of extracellular Ca^{2+} (2 mM). Only myocytes subjected to SR Ca^{2+} depletion exhibited Ca^{2+} entry. Results are representative of n=24 and n=12 CPVT myocytes with depleted and intact, respectively, SR Ca^{2+} stores.

Supplemental Figure 2.

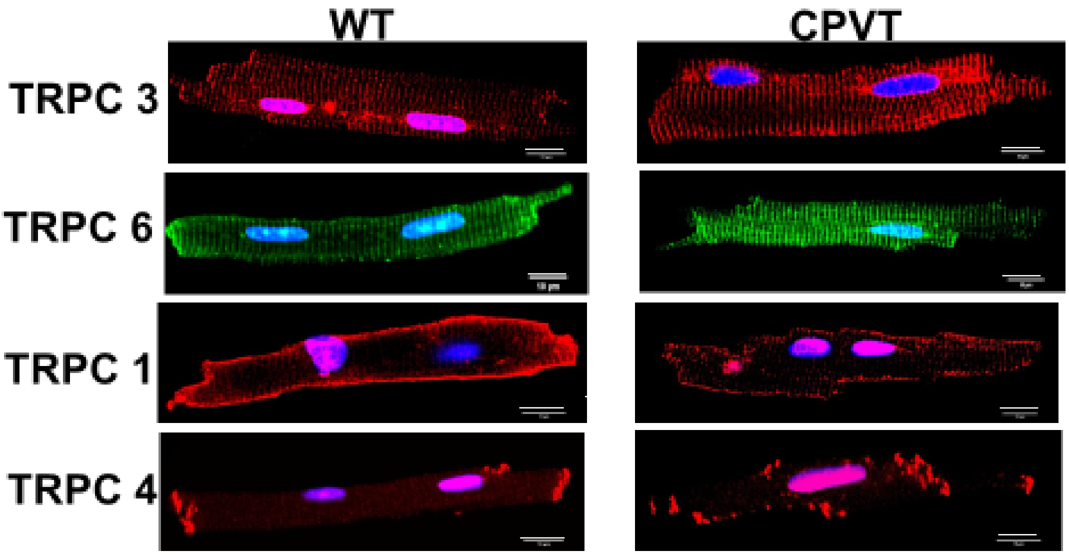

Supplemental Figure 2. Immunohistochemistry confocal images of WT and CPVT myocytes stained for TRPC3, TRPC6, TRPC1 and TRPC4. Scale represent 10 µm.

Supplemental Figure 3

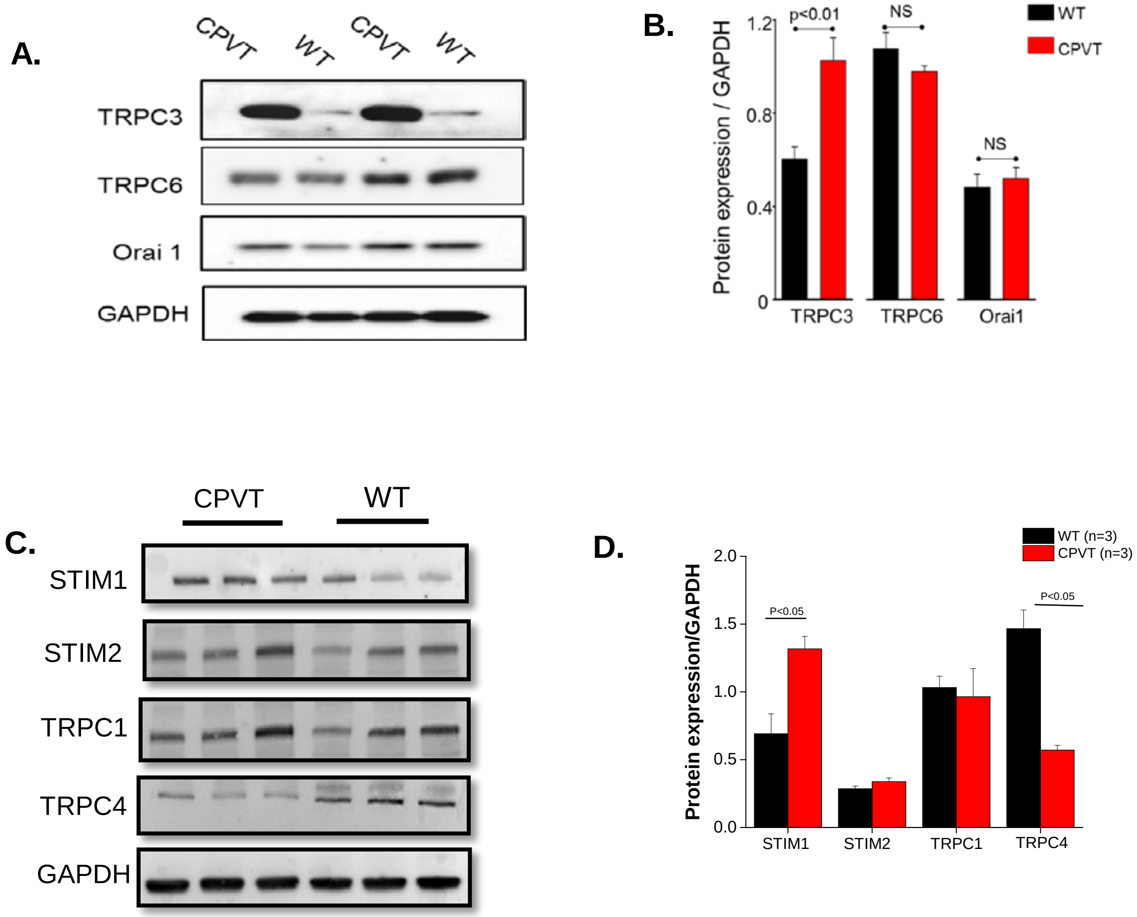

Supplemental Figure 3. A. Representative Western blots of WT and CPVT mouse hearts (50 µg) probing for TRPC3 (n=8), TRPC6 (n=4) and ORAI 1 (n=6), and GAPDH. B. Densitometry of TRPC3, TRPC6, ORAI 1, normalized by GAPDH. A significant increase in TRPC3 is seen from CPVT hearts compared to WT. C. Representative Western blots of WT and CPVT mouse hearts probing for STIM1 (n=3), STIM2 (n=3), TRPC1 (n=3) and TRPC4 (n=3). D. Densitometry of STIM1, TRPC1 STIM2 and TRPC4 normalized to GAPDH, respectively. STIM1 is significantly increased in CPVT hearts compared to WT. TRPC4 was significantly decreased compared to WT hearts. Mean ± SEM tested by t-test ($p < 0.05$).

Supplemental Figure 4.

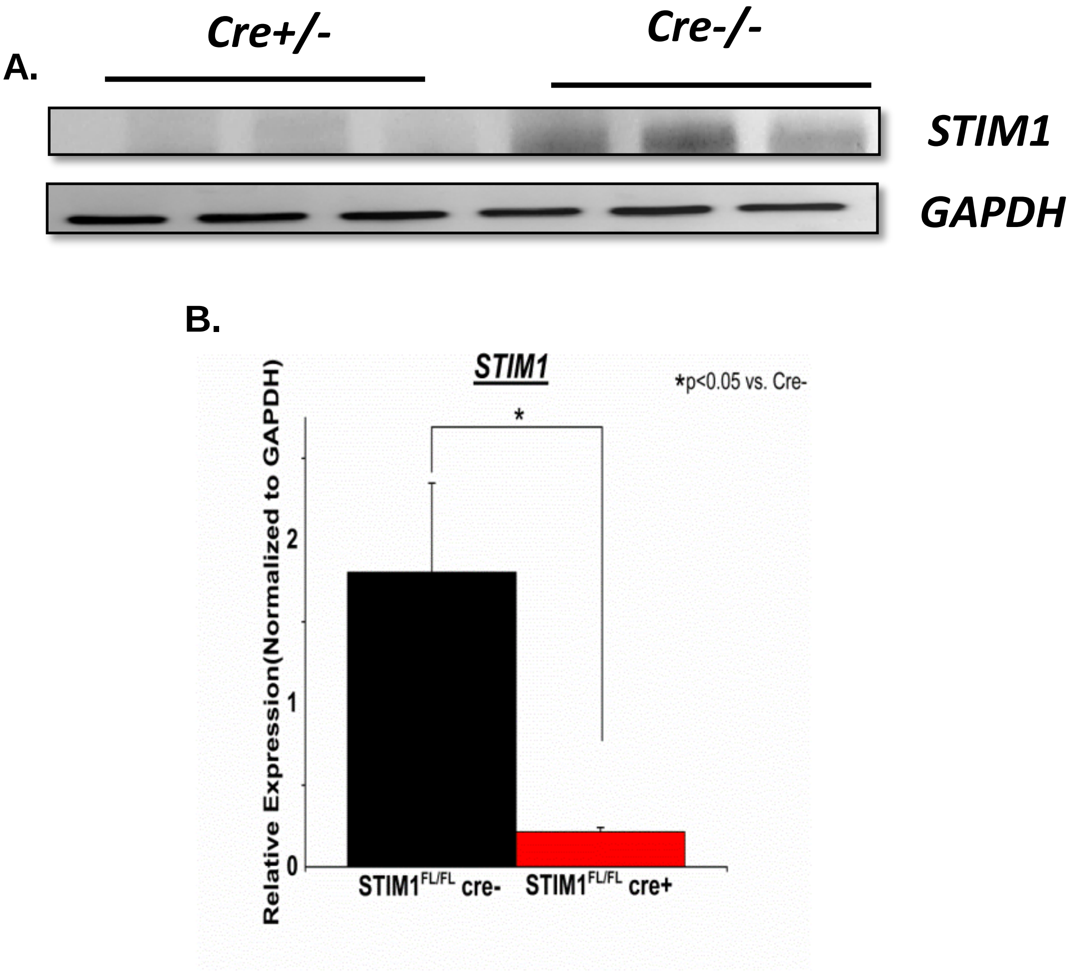

Supplemental Figure 4. cSTIM1KO ($STIM1^{Fl/FL}$ Cre+) mouse hearts show a significant reduction in STIM1 protein compared to control ($STIM1^{Fl/FL}$ Cre+). A. Representative Western blot of control and cSTIM1 KO hearts probing for STIM1 and GAPHD. B. STIM1 protein expression relative to GAPDH. Mean ± SEM tested by t-test ($p < 0.05$).

Supplemental Figure 5.

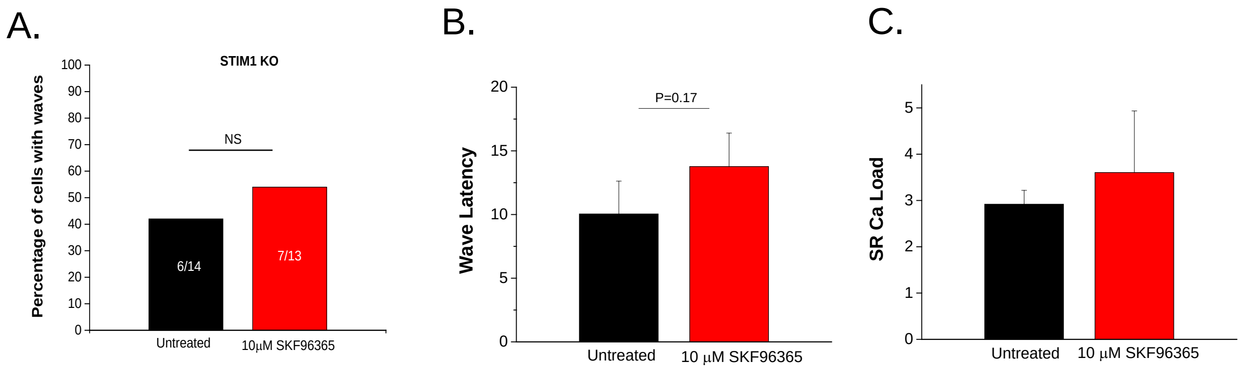

Supplemental Figure 5. SKF95365 (10μM) has no effect on spontaneous Ca^{2+} waves in cSTIM1 KO cardiomyocytes. Fraction of cells exhibiting Ca^{2+} waves (A), average Ca^{2+} wave latency (B) and SR Ca^{2+} content were no different between SKF-treated and untreated cSTIM1KO myocytes. Data are presented as Mean ± SEM tested by t-test ($p < 0.05$).

Supplemental Figure 6.

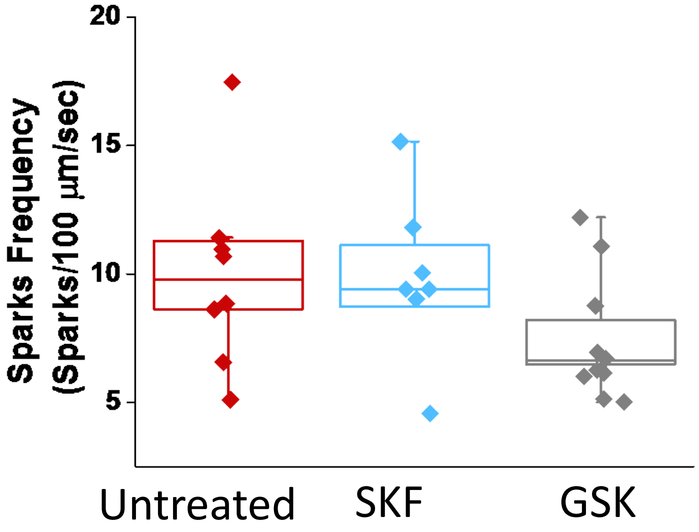

Supplemental Figure 6. SKF96365 (10μM) and GSK7975A (10μM) had no effect on spark frequency in CPVT myocytes. Data are presented as Mean ± SEM tested by t-test ($p < 0.05$).
